# Wheat Rust Diseases: Global Impact, Integrated Disease Management, and Emerging Biotechnological Innovations for Sustainable Crop Protection

**DOI:** 10.3390/biology15191756

**Published:** 2026-10-02

**Authors:** Mingzheng Duan, Muhammad Asif Shabbir, Mustansar Mubeen, Yasir Iftikhar, Naureen Anwar, Shiping Jiang, Zhifang He, Sirui Han, Ahmed Mahmoud Ismail, Khalid M. Alsyaad, Ahmed Ezzat Ahmed, Muhammad Umer

**Affiliations:** 1 Advanced Institute of Ecological Agriculture and Biodiversity on the Yunnan-Guizhou Plateau/Yunnan Key Laboratory of Smart Villages and Agri- Cultural- Tourism Integration, Zhaotong University, Zhaotong 657000, China; duanmingzheng@ztu.edu.cn (M.D.); shipingjiang2004@163.com (S.J.); 202215040106@stu.ztu.edu.cn (Z.H.); 202315120126@stu.ztu.edu.cn (S.H.); 2 Department of Plant Pathology, College of Agriculture, University of Sargodha, Sargodha 40100, Pakistan; mustansar01@yahoo.com (M.M.); yasir.iftikhar@uos.edu.pk (Y.I.); 3 Department of Biological Sciences, Faculty of Science and Technology, Virtual University of Pakistan, Raiwind Road Campus, Lahore 54670, Pakistan; naureen.anwar@vu.edu.pk; 4 Pests and Plant Diseases Unit, College of Agricultural and Food Sciences, King Faisal University, Al-Ahsa 31982, Saudi Arabia; amismail@kfu.edu.sa; 5 Department of Biology, College of Science, King Khalid University, Abha 61413, Saudi Arabia; alsyaad55@hotmail.com (K.M.A.); aabdelrahman@kku.edu.sa (A.E.A.); 6 Prince Sultan Bin Abdelaziz for Environmental Research and Natural Resources Sustainability Center, King Khalid University, Abha 61421, Saudi Arabia; 7 School of Breeding and Multiplication (Sanya Institute of Breeding and Multiplication), College of Tropical Agriculture and Forestry, Hainan University, Sanya 572025, China

**Keywords:** *Puccinia triticina*, yellow rust epidemiology, durable resistance, integrated disease management, precision agriculture, climate change adaptation, global food security

## Abstract

Wheat (*Triticum aestivum* L.) remains one of the most important staple crops worldwide and serves as a primary source of calories and protein for billions of people. Global wheat production, however, continues to be threatened by rust diseases caused by *Puccinia triticina, Puccinia striiformis* f. sp. *tritici* and *Puccinia graminis* f. sp. *tritici*. These pathogens are highly adaptive and frequently give rise to new virulent races, causing resistance in wheat cultivars to breakdown. Under favorable environmental conditions, severe epidemics can develop and substantial yield losses may occur. In response to these challenges, traditional management approaches such as resistant cultivars, crop rotation, sanitation and fungicide application have long been employed in wheat production systems. Yet these strategies have increasingly been constrained by resistance breakdown, genetic uniformity in modern cultivars, and climate-driven changes in pathogen distribution. Conventional management practices are evaluated alongside emerging technological solutions including genomic-assisted breeding, gene pyramiding, CRISPR-based genome editing, biological control agents and precision agriculture tools. In addition, nanotechnology has emerged as a promising tool in plant disease management, where nanoparticles such as silver, copper and zinc oxide exhibit strong antifungal activity and enable targeted delivery of agrochemicals. Nano-enabled formulations can enhance fungicide efficiency, reduce chemical inputs and improve plant defense responses, offering a sustainable complement to existing wheat rust management strategies. Particular attention is given to integrated disease management strategies, which combine traditional agronomic practices with modern surveillance technologies such as remote sensing, artificial intelligence-based forecasting models, and pathogen genomics. These combined approaches can improve resistance durability and build more resilient wheat production systems. Future directions emphasize climate adaptation, global surveillance and collaborative breeding. In an era of increasing climatic uncertainty and evolving pathogen populations, durable rust management will depend on coordinated global efforts that align scientific innovation, farmer adoption, and policy support to safeguard wheat productivity and global food security.

## Simple Summary

Wheat feeds a large share of the world’s people, yet every growing season it faces a persistent enemy: a group of fungal diseases known as rusts. These fungi travel long distances on the wind, multiply quickly, and keep changing in ways that allow them to defeat the protection bred into wheat plants. When conditions favour them, they can ruin much of a harvest, and the damage falls hardest on farmers with the fewest resources. In this article, we review how these diseases have been fought in the past, through resistant wheat varieties, crop rotation, field hygiene, and chemical sprays, and we look at newer approaches that are changing the field. These include breeding guided by the wheat plant’s genetic makeup, targeted gene adjustments, beneficial microbes that keep the fungus in check, and ultra-small particles designed to improve treatment while reducing chemical use. We also describe tools that spot outbreaks early using aerial imaging and computer-based prediction. We aim to show how combining trusted and emerging methods, alongside stronger cooperation among researchers, farmers, and decision-makers, can protect wheat and help secure the world’s food supply.

## 1. Introduction

Wheat (*Triticum aestivum* L.) is one of the most significant staple food crops in the world. With its large genetic diversity and adaptability, it is grown in a wide range of agro-ecological zones from temperate to semi-arid regions. Wheat contributes about a fifth of global caloric intake, provides a substantial share of protein, and is recognised as a mainstay of human nutrition and socio-economic stability [1]. Ongoing trends in the agricultural sector, particularly in breeding and agronomy, along with the adoption of climate-smart agricultural practices, have increased yields and improved crop tolerance to abiotic stresses, including drought, heat, and poor soil fertility [2,3].

Biotic and abiotic stresses are a constant challenge to wheat productivity. Wheat is susceptible to several important fungal diseases, including powdery mildew, leaf rust (*Puccinia triticina*), stem rust, and smut. Among these challenges, rust diseases represent the greatest threat. Leaf rust is attributed to *Puccinia triticina*, yellow (or stripe) rust to *Puccinia striiformis* f. sp. *tritici,* and stem rust to *Puccinia graminis* f. sp. *tritici*. These obligate fungal pathogens show genetic variation linked to rapid evolution and long-distance dispersal by airborne spores [4]. These pathogens [5] have several features that allow them to overcome wheat resistance and cause widespread epidemics across large wheat regions. Wheat rust significantly impacts crop productivity and economic welfare; under heavy disease pressure, production losses can reach 20 to 50% [6], and in severe cases, total crop loss may occur.

Under moderate disease pressure, yield losses typically range from 5–10%, whereas severe epidemics can cause substantially greater damage [7,8,9]. Annual wheat losses from diseases including rusts are 10–28% globally [10]. Host-specific pathogen races, the fragility of resistance in cultivated varieties, and the growing threats posed by climate change complicate rust control. The rapid emergence of virulent rust isolates such as Ug99 stem rust and Warrior and Kranich yellow rust has rendered many resistance genes useless [4,11]. Climate change is altering wheat-growing conditions across regions. Temperature fluctuations, precipitation variation, and milder winters are broadening the belt of wheat stripe rust disease.

As a consequence, disease can now be found in areas where it was not previously present [3,12]. Classical wheat rust management practices such as resistance breeding, fungicide application, crop rotation, and sanitation and eradication of alternate hosts have successively decreased disease pressure and prevented catastrophic yield losses throughout history. It has become increasingly evident that the extensive deployment of single resistance genes, monoculture and frequent fungicide application have intensified resistance erosion in crops and pathogens [7,13]. These issues are most severe in low-income areas, where limited access to resistant seed, fungicides, and disease monitoring disrupts timely countermeasures and devastates small-scale farmers [14].

Over the last decade, integrated crop management concepts have increasingly turned to modern technological innovations. Genomic selection, multi-gene resistance breeding, CRISPR-based gene editing, biological control agents, and precision agriculture tools, combined with advanced technologies (such as remote sensing, AI, and disease forecasting models), creates new possibilities for sustainable rust management [15]. These sources will help develop resistance durability, reduce environmental footprints, and build resilience to climate variation. This review examines classic and innovative methods for combating wheat rusts, focusing on combining conventional and modern approaches. This review highlights wheat’s global significance, the biology and epidemiology of rust pathogens, and their impact on food security. The review also discusses traditional and novel management strategies, with special attention to genetic resistance, breeding approaches, and biotechnological methods. Finally, it considers possible future directions, including climate adaptation, digital surveillance, and international cooperation, stressing the importance of a joint global approach to secure wheat production in a changing world.

Recently, nanotechnology has gained attention as an emerging approach in plant disease management. Nanoparticles have unique physicochemical properties, such as high surface area and enhanced reactivity [16,17], that allow effective interaction with plant pathogens. Metal and metal oxide nanoparticles, including silver (AgNPs), copper oxide (CuO-NPs) and zinc oxide (ZnO-NPs), have demonstrated antifungal activity against several phytopathogens and show potential for managing wheat rust diseases. Furthermore, nano-based delivery systems can improve the efficiency and reduce the dosage of conventional fungicides [18,19] while minimizing environmental impact. Integrating nanotechnology into existing disease management frameworks offers new opportunities to enhance the durability and sustainability of wheat rust control.

## 2. Overview of Wheat as a Staple Crop

**Figure 1:**
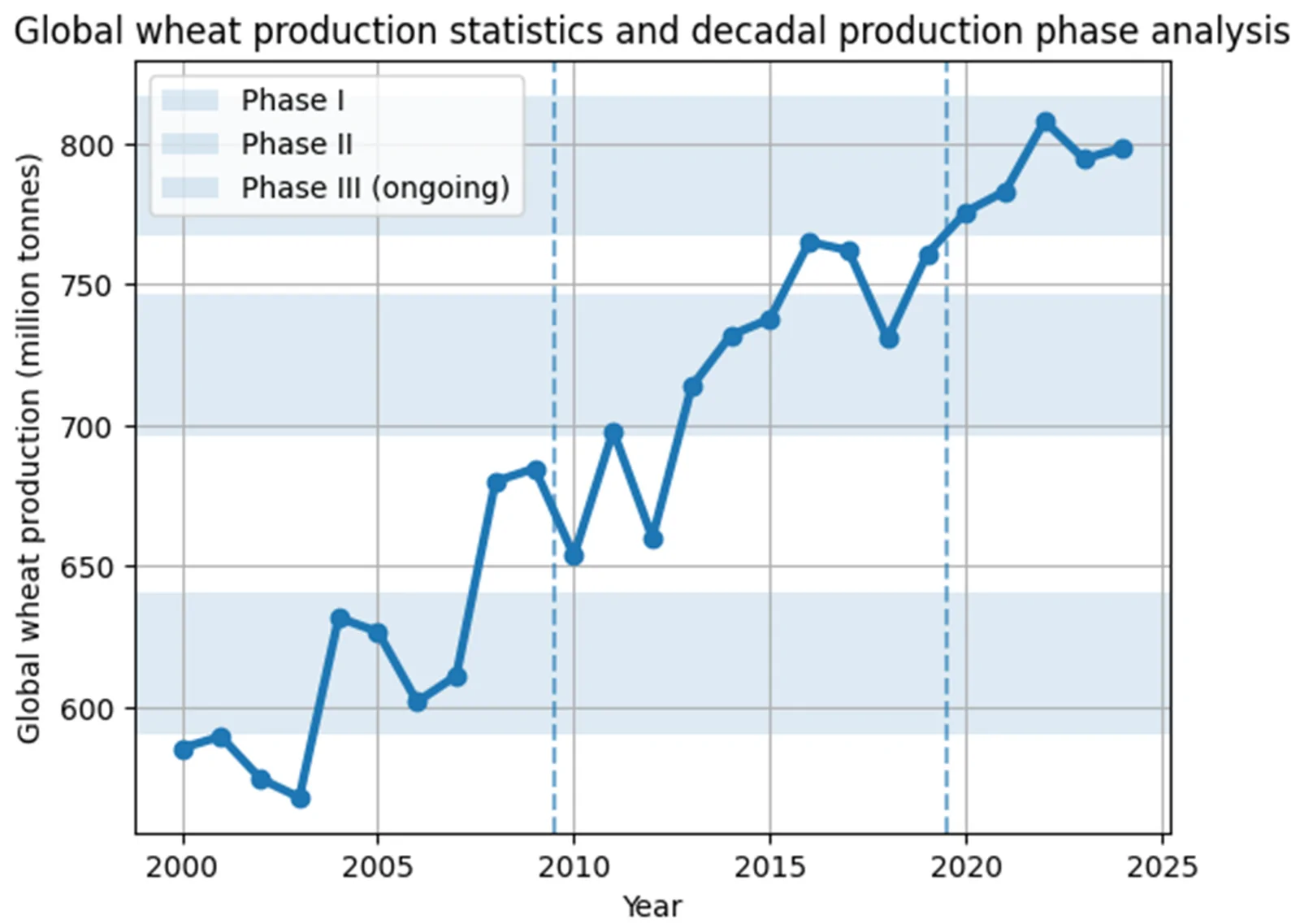
Global wheat production statistics and long-term production analysis by decade (2000–2010, 2011–2020, and the partial decade 2021–2024) [20].

Wheat (*Triticum aestivum* L.) is the most widely grown cereal and a dietary staple for a large share of the world’s population (Figure 1). Global production currently exceeds 780 million metric tonnes annually from about 220 million ha, with China, India, the European Union, Russia and the United States together accounting for more than half of the total [1,20,21]. In recent seasons, China has led with roughly 137 million tonnes, followed by the EU (about 124), India (around 112) and Russia (roughly 92) [20]. Approximately 65–70% of output is used directly as human food and the remainder for feed, seed and industry [22]. At the same time, almost 200 million tonnes are traded annually, making wheat the world’s most traded cereal [23,24]. Because production and exports are concentrated in a few countries, a shock in any one of them can propagate through international markets and raise prices, with the sharpest effects on import-dependent nations in North Africa, the Middle East and sub-Saharan Africa [21,23]; nearly 40% of the global wheat area, moreover, lies in rainfed and semi-arid systems that are especially exposed to climatic and biotic stress [25]. Against this backdrop, wheat rust diseases directly threaten supply stability. Under favourable conditions, epidemics can rapidly reduce national production, with documented losses ranging from 5–10% under moderate pressure to 20–50%, and up to complete crop failure in severe outbreaks [6,7,8,9]; globally, rusts account for a major share of the estimated 10–28% of annual wheat production lost to disease [10]. These quantitative links between production, trade and rust-driven losses show that food security depends not only on expanding output but on protecting existing yields from recurrent, transboundary epidemics.

Beyond total output, wheat’s value lies in the quantity and quality of the grain it delivers: it supplies about 20% of the calories and a comparable share of the protein in the human diet, and whole-grain wheat is an important source of dietary fibre, B-vitamins and minerals such as iron and zinc [26,27,28,29,30]. Grown across a wide range of agro-ecological zones, wheat underpins food and nutritional security in both high- and low-input systems [2,3]. Rust diseases threaten this contribution on two fronts [31,32,33]. First, by limiting photosynthesis and accelerating senescence, they reduce grain number and grain filling, so that severe epidemics translate directly into lower yields [9,34]. Second, the same processes impair grain quality—producing shriveled, low-test-weight grain with reduced protein and starch accumulation—and thereby lower the nutritional and market value of the harvest, even when yield loss is only partial [8,9]. The burden falls hardest on resource-poor farmers, for whom limited access to resistant seed, fungicides and advisory services turns rust outbreaks into direct threats to income and food security [14]. Quantifying and protecting the links between production, yield, and grain quality on the one hand and rust-driven losses on the other is therefore central to sustaining wheat’s role in global food security, and provides the rationale for the integrated management and biotechnological strategies reviewed below.

## 3. Significance of Wheat Rust Diseases

Wheat rusts are among the most devastating and economically important cereal diseases worldwide. Primarily, these diseases are caused by *Puccinia triticina* (leaf rust) and *Puccinia striiformis* f. sp. *tritici* (stripe, yellow rust) and *Puccinia graminis* f. sp. *tritici* (stem rust). These obligate biotrophic fungi continue to challenge wheat production worldwide in all major wheat-growing regions [5]. Their importance stems from their rapid evolution, long-distance dispersal, ability to overcome host resistance, and the enormous yield and quality losses they cause, particularly under favorable conditions. Wheat is affected by a range of economically important diseases, and rusts are best understood within this broader context. Other major fungal and related pathogens, including *Fusarium graminearum* (Fusarium head blight), *Zymoseptoria tritici* (Septoria tritici blotch), the smuts and bunts caused by *Ustilago* and *Tilletia* spp., powdery mildew, and the emerging threat of wheat blast (*Magnaporthe oryzae* pathotype *Triticum*)—also cause substantial regional losses and, in some cases, serious grain quality and food-safety concerns [10,25]. Among this disease complex, however, rusts are distinguished by their capacity for explosive, polycyclic epidemics, long-distance aerial dispersal across continents and rapid virulence evolution, which together make them a recurrent, transboundary and historically catastrophic threat to global wheat production. On this basis, the present review focuses on rusts, while recognizing that durable wheat health ultimately depends on managing the full spectrum of diseases.

### 3.1. Yield Losses and Production Impacts

Grain yield declines directly through limited photosynthesis, premature defoliation and senescence [9,34], stem weakening, and reduced translocation of nutrients to developing grains. Leaf rust infection reduces photosynthetic rate and grain filling, and it also infects leaf undersides; stripe rust leads to earlier leaf death under cool, wet conditions. Stem rust is the most damaging, attacking stems or spikes, causing lodging and up to 100% yield loss in severe epidemics [9]. Documented yield losses vary with disease, cultivar susceptibility and environment, but concrete regional estimates illustrate their magnitude. Under moderate epidemic conditions, losses generally range from 5–10%, rising to 20–50% in severe epidemics when highly susceptible cultivars are grown over large areas, and reaching 70–100% under stem rust pandemics driven by highly virulent races [8,11]. In the high-yielding Indo-Gangetic Plains of South Asia, stripe and leaf rust epidemics have caused recurrent losses in susceptible cultivars, while in China stripe rust remains among the most economically damaging diseases, with commercial cultivar losses closely tracking resistance levels [6]. In North America and Australia, sporadic but severe outbreaks have produced losses of hundreds of millions of dollars through combined yield reduction and fungicide costs [9,35]. Globally, wheat rusts are estimated to account for a major share of the 10–28% of annual production lost to disease in susceptible or unmanaged systems [5,10].

### 3.2. Economic Consequences

Economic losses from wheat rusts extend beyond yield reductions; they also increase production costs for farmers, reduce grain quality, and raise financial risk in wheat-growing areas. Wheat production costs rise significantly because of fungicide applications, seed replacement, disease surveillance, and breeding programs in regions with recurrent rust epidemics [13]. Worldwide, annual economic losses from stripe rust alone amount to more than US$0.9 billion, or the equivalent of approximately 5–6 million tons of wheat grain lost annually [36,37]. Rust epidemics in developed wheat-growing regions, e.g., North America and Australia, have caused hundreds of millions of dollars in annual losses due to decreased yields and increased fungicide applications [35]. The economic burden also differs sharply between large-scale and smallholder systems. In large-scale commercial systems, such as those in North America, Australia, and Western Europe, losses mainly arise from yield reductions and the rising cost of repeated fungicide applications and surveillance, which erode profit margins even when epidemics are contained [13,35]. In smallholder and low-income systems, by contrast, the effects on food security can be far more severe than the direct loss of farm income, because resource-poor farmers frequently lack timely access to fungicides, resistant cultivars, and advisory services. As a result, rust epidemics may deepen rural poverty and undermine livelihood sustainability in already vulnerable agricultural societies, as documented in parts of East Africa and South Asia [14,38].

### 3.3. Threats to Food Security and Market Stability

Wheat rust diseases also present a substantial challenge to national and international food security. Rusts can cause enormous economic losses because large-scale epidemics can quickly disrupt wheat supplies, domestic markets, and food prices. Because wheat is the world’s most traded cereal commodity, production shocks in major exporting regions can cascade through international markets and undermine food availability in import-dependent countries [21]. Global wheat trade is concentrated in a few major exporters, including Russia, the United States, Canada, Australia, and the European Union, so a rust-driven production shortfall or a precautionary export restriction in any one of them can tighten global supply and elevate prices, with the sharpest consequences for import-reliant nations in North Africa, the Middle East, and sub-Saharan Africa [21]. This price transmission shows that the market impact of rust epidemics extends well beyond affected fields, linking regional disease outbreaks to international food security and price stability. Past and present rust pandemics illustrate the potential severity of food crises following such outbreaks. For instance, the Ug99 stem rust lineage has garnered global attention and concern because it can overcome many commonly used resistance genes [39] and threatens wheat-growing areas that supply much of the world’s trade in this crop [11]. The rapid spread of virulent stripe rust races suited to warmer climates has increased pressure in Asia, Europe, and North Africa to the point that production stability in regions essential to global wheat supply is compromised.

### 3.4. Biological Characteristics Underpinning Disease Severity

Wheat rusts are particularly important because of specific biological and epidemiological properties. Rust fungi produce large quantities of airborne urediniospores that can be carried over thousands of kilometers by air currents, so transboundary spread occurs rapidly [4]. Their polycyclic nature permits several infection cycles in a single growing season, resulting in a geometric increase in disease severity under suitable conditions [29]. The great genetic diversity of rust pathogens arises through three complementary mechanisms, each contributing differently to the emergence of new virulent races. Mutation generates novel virulence alleles within clonal urediniospore lineages during repeated asexual cycles on wheat, and because rust populations are enormous, even rare mutations can be selected rapidly under pressure from widely deployed resistance genes. Somatic (parasexual) recombination allows the exchange of genetic material between nuclei of different genotypes without a sexual cycle, occasionally producing new virulence combinations on wheat itself. Sexual reproduction on alternate hosts, most notably *Berberis* spp. for stem rust, permits genetic recombination that can assemble entirely new virulence profiles in a single generation. Together, these processes enable the pathogen to overcome resistance genes soon after they are introduced [40,41,42]. However, this evolutionary plasticity can limit the sustainability of resistance-based control strategies and requires constant surveillance and breeding.

### 3.5. Climate Change and Increasing Disease Risk

Wheat rust diseases are becoming more important due to climate change and its effects on pathogen survival, multiplication, and distribution. For rust diseases, higher temperatures, wider humidity ranges, and milder winters favor earlier disease appearance, longer infection periods and range extensions into areas that were once unfavorably cool [12,26]. Each pathogen, however, has a distinct thermal optimum that shapes where and when it causes greatest severity: leaf rust develops most rapidly at 15–22 °C, stripe rust historically favored cooler conditions of 9–15 °C, and stem rust is most damaging at 20–30 °C [43,44]. Warming is especially significant for stripe rust, because high-temperature-adapted races now cause severe epidemics well above the pathogen’s traditional thermal range, extending its reach into warmer wheat-growing regions previously considered unsuitable [4,45,46]. Stripe rust, historically favored in cool climates, has now found refuge in warmer agro-ecosystems and popular fields across South Asia, Central Asia and some parts of Europe [46]. Climate-driven changes in rust epidemiology create uncertainty and challenges for disease prediction and management [47]. As wheat production becomes more intensive and climate variation increases, the risk of a widespread rust epidemic is expected to rise, further emphasizing the need for well-coordinated, integrated rust management approaches [3].

## 4. Current Challenges in Wheat Rust Management

Wheat rust diseases remain one of the most difficult and aggressive threats to global wheat production, despite significant progress in developing resistant cultivars, disease monitoring, and crop protection technologies. The quest for durable resistance is motivated not only by the rapid evolution of rust pathogens but also by a changing climate with increasing climate variability [5]. Host resistance erosion and socio-economic factors constrain effective disease management, especially in developing countries [5]. These interrelated challenges have intensified the occurrence, severity, and unpredictability of rust epidemics in recent times.

### 4.1. Emergence of New Pathogenic Races

**Figure 2:**
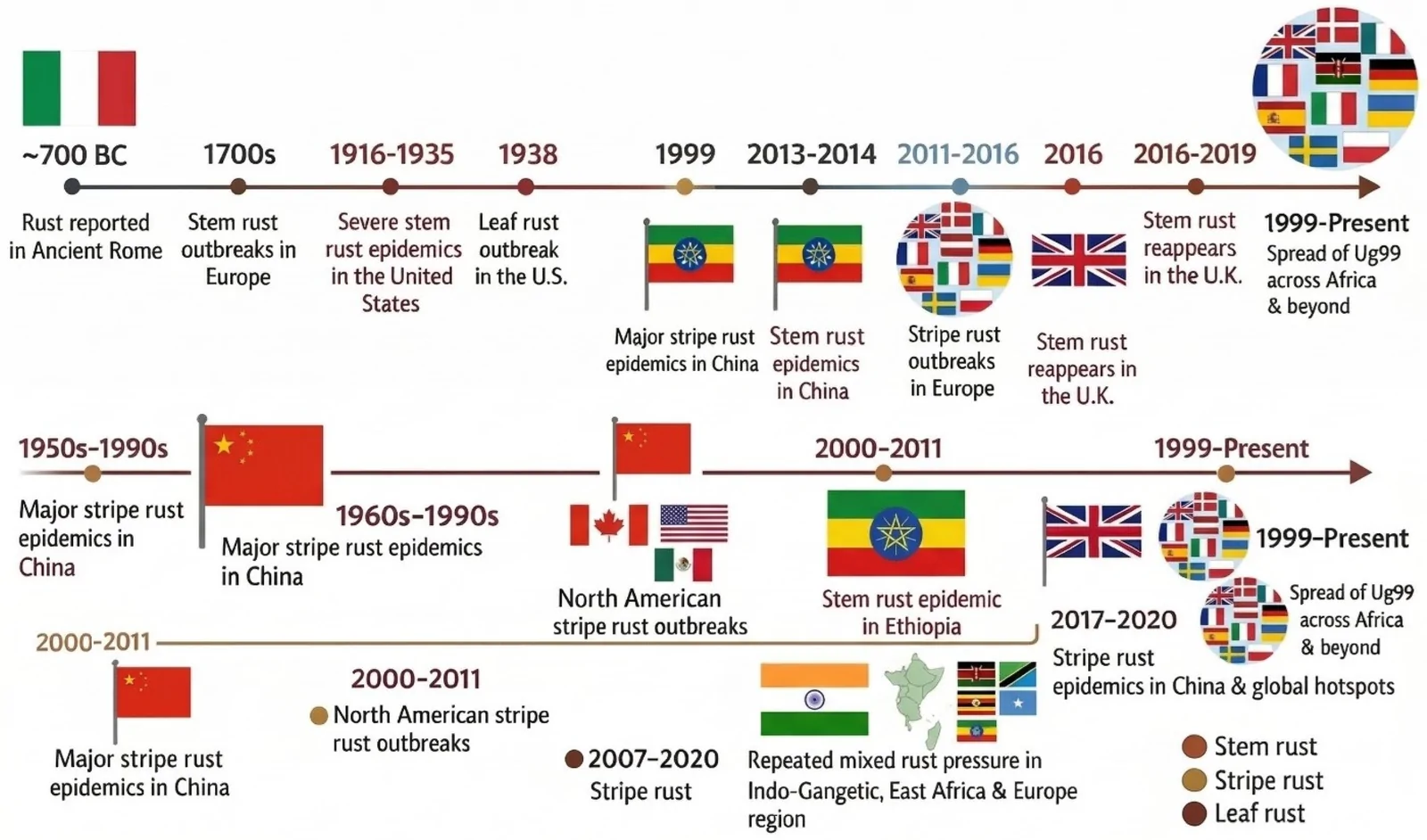
Global distribution of wheat rust epidemics and surveillance with the passage of time.

One major problem in wheat rust control is effectively managing newly virulent pathogen races that can overcome resistance genes. Rust fungi can evolve rapidly because they have large populations, somatic recombination, sexual reproduction on alternate hosts (e.g., Berberis species), and very rapid mutation rates [41]. The dynamic virulence systems they produce can create new combinations too rapidly for widely used resistance sources. Researchers first reported *Puccinia graminis f. sp. tritici* in Uganda in 1999. This lineage has given rise to more than 15 variants that together break over 80 percent of the known stem rust resistance genes deployed in global wheat breeding programs [5,11]. Similarly, highly virulent stripe rust races such as Warrior, Kranich, and PstS13/PstS14 have invaded Europe, Asia, and North Africa [4,45]. Susceptibility is not uniform across wheat cultivars but depends on the specific resistance genes each carries and whether the prevailing pathogen races can overcome them. A cultivar resistant to one race may be fully susceptible to another so that the same variety can perform very differently across regions and seasons as the virulence spectrum shifts; for example, once widely grown cultivars such as PBW 343 in South Asia and Inqalab-91 in Pakistan were initially resistant but became susceptible following the emergence of new races [28,48]. Global and regional pathogenicity surveys, including surveillance of stem rust in Africa and Europe and of stripe rust across Asia and Europe, continue to document this cultivar-by-race specificity [11,42,49]. The rapid turnover of virulent races therefore places constant pressure on breeding programs and shortens the effective lifespan of resistant varieties. Figure 2 summarizes occurrence records, surveillance reports, and climatic suitability assessments for the three rusts worldwide [4,11,27,43].

The present-day virulence landscape: Relative to two decades ago, the dominant virulence profiles of all three rusts have shifted substantially, and this current composition rather than historical epidemics now shapes breeding priorities. In stem rust, the *Puccinia graminis* population is presently defined by the Ug99 lineage and its more than fifteen derived variants, together with independently evolved races such as TKTTF, which collectively defeat a large proportion of deployed Sr genes, including the once broadly effective Sr31 [5,11]. In stripe rust, the populations that prevailed before 2011 have been largely displaced across Europe and Asia by the aggressive, high-temperature-adapted Warrior, Kranich and PstS13/PstS14 lineages, rendering many previously effective Yr genes ineffective [4,45]. In leaf rust, continual mutation has progressively eroded the value of major genes such as Lr1, Lr13 and Lr26 in many regions [42,50]. For modern breeding programs, the central implication is that reliance on individual race-specific genes is increasingly short-lived: durable protection now depends on combining pleiotropic adult-plant resistance genes such as Lr34/Yr18/Sr57 and Sr2 with region-specific gene stacks matched to the prevailing virulence spectrum, guided by continuous virulence surveillance [5,51].

### 4.2. Climate Change and Geographic Expansion of Rust Diseases

Climate change has been identified as a significant factor modifying wheat rust disease epidemiology. Increased temperatures, changing rainfall patterns, prolonged dew periods, and milder winters increase pathogen survival and speed up the infection cycle, which subsequently helps rust diseases move into new areas [12]. Geographic expansion occurs through several complementary routes: long-distance dispersal of urediniospores on wind currents that seed new regions, thermal adaptation of pathogen races to previously unsuitable climates, northward and upslope shifts in climatically suitable zones, and inadvertent movement of infected plant material through trade [4,12]. Through these routes, yellow rust, traditionally confined to cool, humid areas, has extended into warmer regions of South Asia, Central Asia, and southern Europe. In contrast, stem rust races such as the Ug99 lineage have moved from East Africa toward the Middle East and beyond [11,43,46]. Climate-induced reshuffling also complicates disease forecasts and responses. By the time symptoms appear, traditional crop-sowing windows and fungicide application schedules are too late. Modeling efforts suggest that in a future climate, areas susceptible to rust will move northward and toward higher elevations, likely increasing disease pressure in regions with less historical exposure to rust management [3,52]. These changes call for climate-smart disease management approaches to combine epidemiological modeling with adaptive agronomy.

### 4.3. Resistance Breakdown and Genetic Bottlenecks

The crop has also been planted with a narrow genetic diversity of major resistance genes, which has led to further genetic bottlenecks and, ultimately, rapid allele spread within the population, often leading to rapid breakdown. Genes including Sr31, Yr27, and Lr26 were deployed across wide production areas for a long time, resulting in consistent selection pressure that promoted the co-evolution of avirulence in rust populations [42,51]. Many modern high-yielding wheat cultivars have narrower genetic bases than traditional landraces and wild relatives, thereby restricting access to novel sources of resistance for breeding. This homogeneity compromises the long-term durability of resistance and makes the crop prone to new rust races [48]. Although race-specific, gene-for-gene resistance is highly effective initially, it is rarely durable, because the pathogen quickly evolves matching virulence. Overcoming this genetic bottleneck will therefore depend on broadening the resistance base with genes that confer more durable, broad-spectrum protection. Pleiotropic adult-plant resistance genes such as *Lr34/Yr18/Sr57*, *Lr46/Yr29* and *Sr2*, which act against multiple rusts and have remained effective across decades and environments, together with newly characterized loci recovered from landraces and wild relatives, represent particularly promising targets for future deployment [45,51,53]. Combining (stacking) such genes with race-specific resistance offers a route to sufficient and more durable disease control under high pressure.

### 4.4. Inadequate Surveillance and Rapid Transboundary Spread

Wheat rust pathogens produce vast numbers of aerially dispersed urediniospores that can be carried thousands of kilometers across continents within days, enabling rapid transboundary movement [4]. Transcontinental transmission typically follows prevailing wind trajectories, as atmospheric dispersal modeling has traced spore movement from East Africa into the Arabian Peninsula and South Asia; it can also occur through inadvertent transport of infected plant material along trade routes [4,26]. Effective surveillance to detect this movement relies on a combination of field-based disease scouting, spore-trap networks, race typing through phenotyping on differential wheat lines, and increasingly molecular and genomic diagnostics that identify virulence changes, coordinated through international platforms such as the Global Cereal Rust Monitoring System and the Wheat Rust Toolbox [5,54]. However, wheat-growing areas in much of Africa and parts of Asia still lack robust surveillance systems, diagnostic capacity, and trained personnel [27]. Sound policy-making for management therefore relies on early monitoring, surveillance, and response actions at the regional level. However, wheat-growing areas in much of the world, especially Africa and parts of Asia, lack strong surveillance and diagnostic systems and trained personnel [27]. Even with the help of international efforts such as the Borlaug Global Rust Initiative (BGRI) and the Global Cereal Rust Monitoring System, pathogen surveillance and data sharing are partial and too often reactive in many parts of the world [5]. Delays in race identification and communication can allow epidemics to develop before management actions are implemented, resulting in significant yield losses.

### 4.5. Socio-Economic and Policy Constraints

Farmers’ socio-economic characteristics greatly determine the success of wheat rust control. These constraints are most acute in the wheat-growing regions of sub-Saharan Africa, particularly the Ethiopian highlands and East Africa, and in parts of South and Central Asia, where smallholder farmers frequently lack ready access to resistant cultivars, affordable fungicides, extension and training services, and credit, limiting their capacity to respond to rust outbreaks [14,38]. In these same regions, weak seed-delivery systems and slow varietal turnover mean that newly released resistant cultivars often reach farmers only after long delays, eroding the benefits of breeding gains at the farm level [38]. The high cost of fungicides, combined with uncertainty in disease predictability, deters timely treatment and leaves plants susceptible to severe epidemics. Policy shortcomings worsen these challenges, including under-investment in agricultural research and problematic seed delivery systems. In most areas, delays in releasing and widely distributing new resistant varieties can erode the benefits of breeding gains at the farm level [38]. Solving the problem of wheat rust requires a combination of scientific innovation, supportive policies, institutional capacity development, and equitable delivery of technologies.

## 5. Historical Context of Wheat Rust Diseases

Wheat rust diseases have accompanied wheat farming since the dawn of agriculture, repeatedly shaping farming practices, breeding priorities and food-security policies. This long co-evolutionary relationship has made rusts resilient and adaptive threats, and understanding their history is essential for managing these diseases today.

### Evolutionary History and Major Epidemics of Wheat Rust Pathogens

Wheat rust pathogens have co-evolved for millennia with host wild grass–pathogen systems that precede wheat domestication by thousands of years. Molecular phylogenetic analysis indicates that *Puccinia triticina*, *Puccinia striiformis* f. sp. *tritici* and *Puccinia graminis* f. sp. *tritici* developed from wild ancestral rust fungal species infecting uncultivated grasses of the Fertile Crescent and nearby areas in which wheat was first domesticated [32,49]. As wheat cultivation spread geographically, so did rust pathogens, with their hosts co-adapted to new environments and wheat genotypes. Rust fungi have complex life cycles involving multiple spore stages and, in some instances, additional hosts that enable sexual recombination. *Puccinia graminis* f. sp. *tritici* is heteroecious, and part of its life cycle occurs on Berberis species, where sexual reproduction produces new genetic combinations and increased virulence variation [40]. This recombination potential is a significant factor in the development of new races that can overcome resistance genes in wheat. While alternate hosts for leaf and stripe rust are less important, recent findings indicate that related plant species may still maintain these pathogens and increase their diversity in specific scenarios [55]. For centuries, the co-evolutionary stand-off between wheat and rust has driven waves of resistance deployment and breakdown. With each major resistance gene, a new race with matching virulence has emerged, indicating that this host–pathogen interaction remains dynamic and unresolved [5].

Wheat rust infection was mentioned in the earliest written history and frequently caused dramatic agricultural and social upheavals. One of the earliest recollections of a wheat rust epidemic is from Rome, where the disease was so feared that religious rites were offered to the god Robigus, and the festival Robigalia was celebrated to protect crops from it [56]. These early citations remind us that rust was long-recognized as a major agricultural malady. Some of the worst rust epidemics in modern times occurred between the 19th and 20th centuries. In the USA, the 1916 stem rust epidemic led to the loss of 30% of national wheat production, with associated famine and economic hardship [57]. Later epidemics in North America during the 1950s reinforced the susceptibility of wheat production systems and led to massive investments in breeding rust-resistant cultivars. The mid-20th century saw a major shift in rust control with the breeding of high-yielding wheat varieties resistant to rust as part of the Green Revolution. Under Norman Borlaug’s leadership, this work achieved enormous reductions in stem rust losses and contributed to significant increases in world wheat production. This success was, however, short-lived, and dependence on a small number of resistance genes created new vulnerabilities [58]. One of the most significant findings is that the Ug99 race of stem rust first emerged in Uganda in 1999. Ug99 and its progenies escaped Sr31 resistance, which had provided effective protection to a major proportion of the world’s wheat varieties [11]. The rapid spread of Ug99 throughout East Africa, and then into the Middle East, triggered worldwide concern about its potential to cause catastrophic yield losses in key wheat-growing areas. More recent stripe rust outbreaks caused by aggressive races like Warrior and Kranich have also led to significant yield losses in Europe and Asia. These two races have been dominant since 2011, and they are virulent on resistance in most commercial cultivars, increasing reliance on fungicides and breeding efforts [4]. Together, these historical outbreaks have caused billions of dollars in losses, disrupted food supplies worldwide, and reordered national and global wheat research priorities. Beyond their economic toll, recurrent epidemics in regions such as East Africa and South Asia have reduced grain reserves, increased import dependence and strained government budgets through emergency subsidies, revealing wheat rust as a driver of food insecurity and socio-economic upheaval, not merely an agronomic challenge [27]. Figure 2 summarizes the global distribution of these historical and contemporary rust epidemics and the associated surveillance record [4,10,11,12,43].

## 6. Understanding Wheat Rust Diseases

Wheat rust diseases are widespread, chronic fungal diseases that threaten wheat productivity globally. Their significance stems from their destructive power, biological complexity, and ability to evolve rapidly and disperse over long distances. Comprehensive knowledge of their taxonomy, biology, epidemiology, and ecological relationships is a prerequisite to sustainable management.

### 6.1. Disease Overview: Definition and Classification of Wheat Rust Diseases

Wheat rust diseases are caused by obligate biotrophic fungi of the genus *Puccinia* (order Pucciniales, phylum Basidiomycota), which survive only on living host tissue. They infect above-ground plant parts and develop characteristic rust-coloured pustules containing large numbers of readily dispersed spores [9]. Three primary rusts affect wheat worldwide.

The three pathogens differ in infection site and behavior:

- Leaf rust (*Puccinia triticina*) infects mainly the leaf blades and is the most widely distributed of the wheat rusts in terms of host range worldwide [50,59].
- Stripe rust (*Puccinia striiformis* f. sp. *tritici*) occurs in cooler, wetter environments and produces characteristic linear (striped) pustules along the leaf veins [43].
- Stem rust (*Puccinia graminis* f. sp. *tritici*) attacks stems, leaf sheaths, and spikes, rupturing the epidermis, weakening the stem, and promoting lodging, frequently with catastrophic yield loss [9,60].

Nevertheless, these rusts all share reduced photosynthetic efficiency and accelerated senescence and grain filling. Biologically, wheat rust fungi are characterized by intricate life cycles that may comprise five discrete spore forms (urediniospores, teliospores, basidiospores, pycniospores, and aeciospores). These urediniospores are the most epidemiologically significant because they can continue infection cycles within a given growing season, driving epidemic development [55]. Stem rust is heteroecious, requiring *Berberis* spp. as the obligate alternate host to complete its sexual cycle, although asexual urediniospore epidemics on wheat can proceed without it. Sexual recombination on *Berberis* generates new virulence combinations that increase pathogen diversity and accelerate resistance breakdown [40]. Leaf rust and stripe rust are predominantly reproduced asexually on wheat, but scarce sexual recombination is suggested under certain ecologies [50]. Molecular biology has strengthened the classification and monitoring of wheat rust pathogens. Molecular markers, whole-genome sequencing, and race-specific diagnostics are increasingly used to discriminate pathogen lineages, track virulence evolution, and inform resistance breeding efforts [45]. These tools have revealed profound genetic diversity in rust populations and highlighted the dynamic nature of wheat–rust interactions. Globally, wheat rusts cause losses of 15–25% in severe outbreak years and much higher losses when susceptible varieties are grown under favorable ecological conditions [27]. Given their continued threat and evolutionary potential, wheat rusts remain one of the most significant problems in cereal pathology and require coordinated, proactive control.

### 6.2. Prevalence and Distribution: Global Epidemiology of Wheat Rust Diseases

Wheat rust is one of the most widely distributed transboundary plant diseases, affecting a wide range of wheat-growing areas across the globe. The general spread of these diseases is favored by rust fungi’s ability to produce large amounts of aerial spores, survive on alternate or volunteer hosts, and take advantage of favorable environmental conditions for multiple infection cycles [5]. Although leaf rust, stripe rust and stem rust are all transmitted by similar means, each disease has a unique set of epidemiological patterns influenced by climate, host genetics and pathogen evolution.

#### 6.2.1. Global Distribution Patterns

Leaf rust is the most common worldwide and occurs in nearly all wheat-growing areas. It is prevalent in areas with moderate temperatures (15–22 °C) and sufficient moisture, and it remains an enduring challenge in South Asia, East Asia, North America, Europe, and parts of Africa [50]. Because leaf rust can survive on volunteer wheat and other grasses, it often establishes early in the season. It causes chronic yield losses in non-epidemic years. *P. striiformis* f. sp. *tritici* has been known to prefer cool and moist areas and has a limited distribution in highlands or temperate zones. However, over the past two decades, the pathogen has spread to warmer environments in South Asia, Central Asia, North Africa, southern Europe, and parts of Australia [4]. Beyond the Ug99 stem rust lineage, several other virulent races are of major current concern, including the stripe rust races Warrior, Kranich, PstS13 and PstS14, which have spread across Europe, Asia and North Africa, and the stem rust race TKTTF (“Digalu”), which caused a severe epidemic in southern Ethiopia in 2013–14 independently of the Ug99 group [4,11,45,61]. Race-specific virulence in leaf rust populations similarly continues to overcome widely deployed genes such as Lr1, Lr13 and Lr26 [42,50]. Stripe rust epidemics are often explosive, with rapid infection cycles because the pathogen completes its life cycle under long leaf-wetness periods and mild temperatures, causing severe yield loss. Wheat stem rust (*Puccinia graminis* f. sp. *tritici*) was well managed in most regions into the mid-20th century, but has become a renewed threat with the emergence of new virulent races like Ug99 and its lineage. Stem rust remains most important in East Africa, a global center of diversity for the pathogen. Still, it has spread into western Asia and perhaps to parts of Europe and southern Asia [11,49]. The occurrence of *Berberis* spp. in both regions provides an opportunity for sexual recombination and seeds the genetic variation associated with regional virulence.

#### 6.2.2. Regional Epidemiological Hotspots

In South Asia, the Indo-Gangetic Plains of India, Pakistan and Nepal form one of the world’s major wheat–rust hotspots, where stripe rust and leaf rust predominate and are endemic owing to high cropping intensity, favorable cool winters and genetically uniform cultivars, while stem rust remains a latent threat [7]. East Africa, including Ethiopia and Kenya, is dominated by stem rust and is a global diversity hotspot for the pathogen, acting as a source area from which new races spread; stripe rust is also increasingly important in the Ethiopian highlands [49,61]. In recent years, rust pressure in Europe and Central Asia has increased due to warmer winters and the proliferation of aggressive stripe rust races, such as Warrior and Kranich. In Europe and Central Asia, stripe rust predominates through aggressive races such as Warrior and Kranich, with increasingly severe outbreaks reported in the United Kingdom, Germany, Denmark and Kazakhstan, while stem rust has re-emerged in western Europe and is a renewed concern; these outbreaks are often associated with resistance breakdown in commercial cultivars [4,11]. In North America, the Great Plains has continued to experience sporadic yet severe rust outbreaks, while ongoing stripe and leaf rust activity, coupled with climate variability and pathogen evolution, was noted in Australia [11].

#### 6.2.3. Epidemiological Trends and Disease Dynamics

Worldwide epidemiology shows increasing frequency, severity, and unpredictability of wheat rust disease. Disease dynamics have shifted as a result of climate change, including pathogen overwintering, spore production, and epidemic longevity [12,26]. Furthermore, international wheat trade and increased transfer of plant material have enabled long-distance migration of races between continents. Sympatric mixed infections with two or more rust species are being reported frequently, especially in areas like South Asia and East Africa. Simultaneous (“mixed”) infection by leaf and stripe rust can substantially compound yield losses under favorable conditions, exceeding the damage caused by either rust alone [7,50]. It is estimated that 35–40% of global wheat-producing areas are at risk of an economically significant rust epidemic in any year [27], highlighting the need for coordinated global surveillance and management. Recent progress in epidemiological modeling, remote sensing, and molecular race tracking is expanding opportunities to monitor rust distribution and predict epidemics. Resources that combine climate data with spore dispersal models and genomic surveillance are increasingly used to inform early warning systems and area-wide disease management policies [26]. Yet surveillance capacity gaps, especially in developing regions, hinder the impact of global efforts to control rust.

#### 6.2.4. Current Pathogen-Specific Status

Although the three rusts are often discussed collectively, they differ markedly in their present-day distribution, epidemic behaviour and economic weight.

Leaf rust (*Puccinia triticina*) is currently the most widely distributed of the three, occurring in nearly all wheat-growing regions including South Asia, East Asia, Europe, North America and parts of Africa. Although individually the least destructive per epidemic, its high frequency and near-global range make it responsible for the greatest cumulative annual losses, and its populations continue to overcome widely deployed genes such as Lr1, Lr13 and Lr26 [50,59]. Stripe rust (*Puccinia striiformis* f. sp. tritici) has undergone the most pronounced recent change in distribution. High-temperature-adapted, aggressive lineages—notably Warrior, Kranich and the PstS13/PstS14 groups—have become dominant across Europe, Asia and North Africa since around 2011 and have extended the disease into warmer regions once considered unsuitable, making it currently the most dynamic rust in terms of range expansion [4,45]. Stem rust (*Puccinia graminis* f. sp. tritici) is the most potentially destructive but currently the most geographically restricted of the three. Following its mid-twentieth-century control, it has re-emerged as a global concern through the Ug99 lineage and independently evolved races such as TKTTF; East Africa remains its principal diversity hotspot and source region, with incursions reported into the Middle East, western Asia and, more recently, parts of Europe [11,49]. Economically, leaf rust imposes the largest steady cumulative burden through chronic, widespread losses, stripe rust drives the most volatile recent epidemic losses through rapid range expansion, and stem rust represents the greatest catastrophic-risk threat should the Ug99 lineage spread more widely into major production zones.

### 6.3. Types of Wheat Rust Diseases

Based on causal agent, symptom expression, infection sites, and epidemiological aspects, wheat rust diseases are grouped into three major types: leaf rust, stripe (yellow) rust, and stem rust. Although closely related, they differ in biology, environmental requirements, and impact on wheat productivity (Table 1).

#### 6.3.1. Leaf Rust (*Puccinia triticina*)

Leaf rust, caused by *Puccinia triticina*, is one of the most common and widely distributed rust diseases of wheat globally. It infects leaf blades primarily, forming small, round to oval orange-brown pustules (uredinia) generally scattered over the leaf surface. Heavy infections cause early leaf yellowing; the photosynthetic area is reduced, and grain filling is impaired, with grain weight and yield losses usually ranging from 10–30% under favorable growing conditions [50]. Leaf rust thrives under moderate temperatures (15–22 °C) and high relative humidity that generally prevail in wheat-growing areas of South Asia, East Asia, Europe, and North America, as well as parts of Africa [62]. The pathogen is mostly propagated through an asexual cycle based on urediniospore transmission through the air over long distances. *Thalictrum* spp. have been reported as alternate hosts, though their epidemiological role appears limited; they may contribute to epidemic development under field conditions but do not seem to contribute much in most regions [55]. Genetically, P. *triticina* is highly polymorphic, with frequent appearance of additional virulence combinations that can overcome widely used major genes Lr1, Lr13, and Lr26. This fast-evolving pathogen has caused multiple resistance breakdowns in wheat cultivars grown in commercial fields and thus requires monitoring and breeding for durable resistance [42].

#### 6.3.2. Stripe Rust (*Puccinia striiformis* f. sp. *tritici*)

Stripe rust, caused by *Puccinia striiformis* f. sp. *tritici*, is highly aggressive in cooler and wet conditions. The disease is known for yellow to orange pustules that appear as linear stripes on leaf veins, leaf sheaths, and sometimes spikes. The loss of photosynthetic efficiency and early death caused by stripe rust can reduce yield by 20–70% during severe epidemics [22,36,43]. Stripe rust has historically been limited to cool environments (9–15 °C), but it has evolved adaptively, resulting in new races capable of growing at higher temperatures and incursions into the warmer areas of South Asia, North Africa, Europe and Australia [4,45]. This mutation has enabled more frequent and more volatile outbreaks of stripe rust. Stripe rust populations are highly dynamic, with aggressive lineages such as Warrior, Kranich, PstS13, and PstS14 overcoming the resistance genes in most of today’s wheat cultivars. Durability of resistance genes such as Yr5, Yr15, Yr36, and Lr46 is maintained in varieties, but other control methods need to be integrated with the strategic deployment of these genes [46].

#### 6.3.3. Stem Rust (*Puccinia graminis* f. sp. *tritici*)

Stem rust, incited by *Puccinia graminis* f. sp. *tritici*, is the most significant of all wheat rust diseases and has been responsible for devastating reductions in cereal yields. The infection occurs in the stem, leaf sheath, leaves and spikes, where it forms elongated reddish-brown pustules that rupture the epidermis. Severe infections can reduce stem strength, promote lodging, interfere with nutrient translocation, and lead to complete crop loss [9]. Stem rust is favored by warm, humid conditions (20–30 °C). Although many areas largely controlled it in the mid-20th century through resistant varieties and barberry eradication, worldwide concern has revived because of the Ug99 group of races. Ug99 and its variants have defeated many of the most important resistance genes, including Sr31 and Sr24 and pose a significant threat across Africa, the Middle East and parts of Asia [11,49]. Unlike leaf and stripe rust, stem rust is heteroecious and biotrophic on alternate hosts (mostly *Berberis* spp.), which involve sexual reproduction. Sexual recombination on barberry allows the formation of new virulent races and plays a prominent role in enhancing the pathogen’s genetic variation and evolutionary potential [40].

#### 6.3.4. Life Cycles and Dispersal Mechanisms of Wheat Rusts

**Figure 3:**
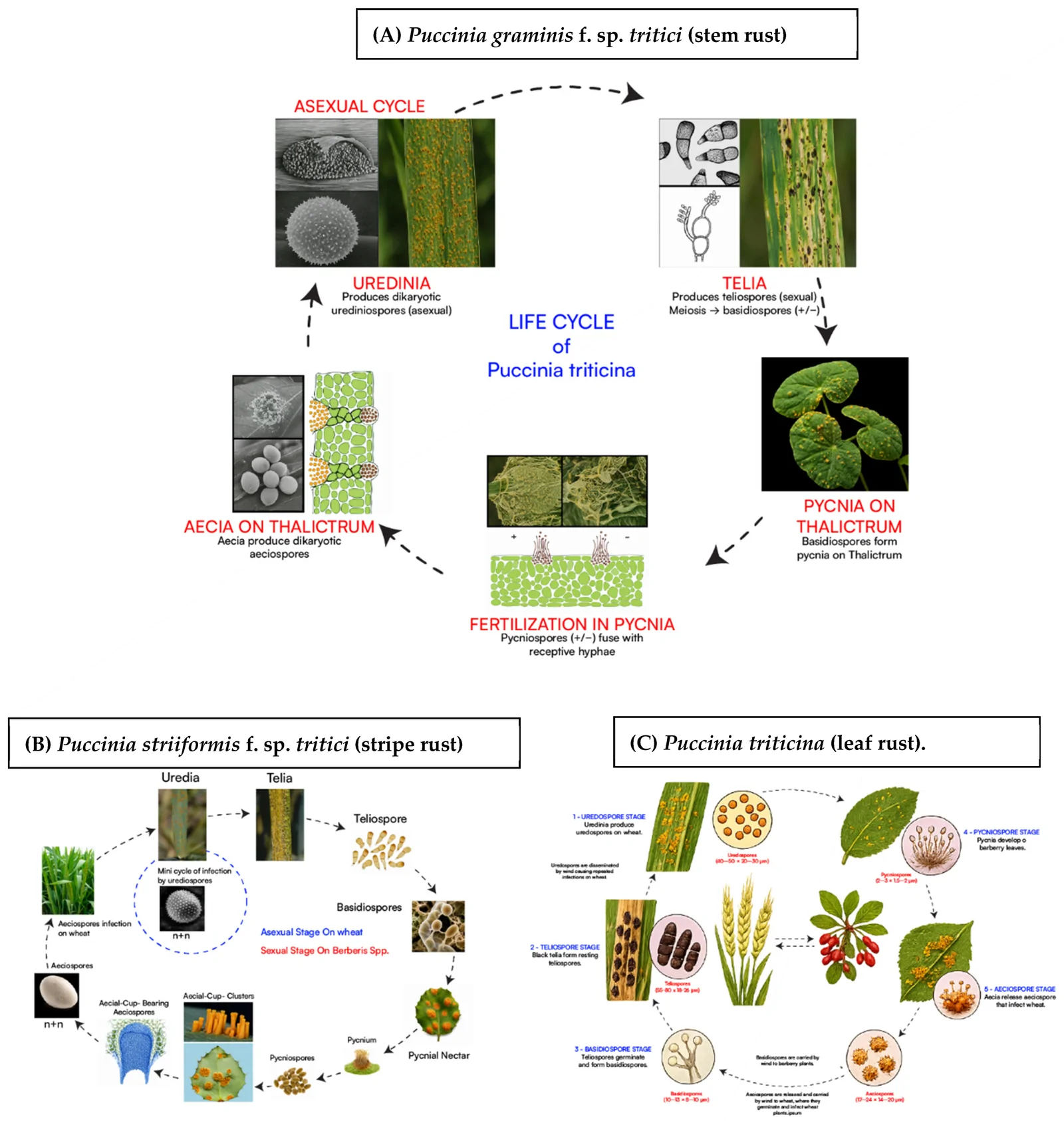
Life cycles and dispersal mechanisms of the three major wheat rust pathogens. (**A**) *Puccinia graminis* f. sp. *tritici* (stem rust), a macrocyclic, heteroecious pathogen with a sexual stage on *Berberis* spp. and successive asexual urediniospore cycles on wheat. (**B**) *Puccinia striiformis* f. sp. *tritici* (stripe/yellow rust), in which sexual reproduction on *Berberis* spp. is regional and epidemics are driven mainly by asexual urediniospores. (**C**) *Puccinia triticina* (leaf rust), with a primarily asexual cycle on wheat; the sexual stage on *Thalictrum* spp. is rare and shown for completeness. Arrows indicate spore stages, host switches, and long-distance dispersal of airborne urediniospores [4,43,59].

All wheat rust pathogens are obligate parasites with a complex life cycle consisting of up to five spore stages: urediniospores, teliospores, basidiospores, pycniospores and aeciospores (Figure 3). Of these, urediniospores are epidemiologically most important because they initiate fast, repeated infection cycles within one growing season [55]. Wind spreads rust spores and can carry them thousands of kilometers between continents, greatly contributing to transboundary disease dispersal. Atmospheric dispersal modeling shows that spores can travel long distances from East Africa into the Middle East and South Asia within weeks, helping explain the rapid global dissemination of virulent races [4,11,26]. Spore germination and infection require high relative humidity (>85%), prolonged leaf wetness and favorable temperatures. Understanding the life cycle and dissemination mechanisms of wheat rust pathogens is essential for establishing efficient forecasting systems, early warning systems, and integrated disease management plans.

### 6.4. Role of Environmental Factors and Alternate Hosts in Wheat Rust Epidemiology

Alternate plant hosts and environmental factors are crucial to the development, spread, and maintenance of wheat rust diseases. Epidemic severity, geographic range, and seasonal cycle are influenced by a combination of climate variables; however, interactions among these, host plant availability, and pathogen biology also affect them. Understanding these factors is important for successful disease prediction and sustainable management.

#### 6.4.1. Environmental Determinants Influencing Disease Epidemiology

Temperature, RH%, leaf wetness duration, and wind conditions are the major factors driving wheat rust infections. All rust pathogens have distinct environmental requirements that affect infection efficiency, spore production and epidemic development. Leaf rust is an obligate parasite that requires a moderately warm temperature between 15 and 22 °C along with high relative humidity (≥80%) for its optimum development. These are the optimum conditions for urediniospore germination and penetration into stomata, resulting in rapid establishment of the disease cycle in multiple temperate wheat regions [34,50,62]. Stripe rust caused by *Puccinia striiformis* f. sp. *tritici* is more tolerant to low temperatures (9–15 °C) and it needs a wet leaf for a long time because colonization capability is greatly reduced when there is no dew in the morning or when weather conditions are cloudy [43]. In contrast, stem rust thrives at higher temperatures (20–30 °C) and is more severe in warm, humid conditions common in tropical and subtropical regions [44]. Wind is a primary driver of rust spread, carrying urediniospores over long distances. Predictive models and field observations show that rust spores can disperse thousands of kilometers in a few days, enabling transboundary spread across continents [4]. Moisture is essential for spore germination, and dew or rainfall on leaves is important, as most infection periods require leaf wetness greater than 6 h for successful infection [63]. Climate change is exacerbating the impact of environmental conditions on rust epidemiology. Increasing average temperatures, shifting rainfall patterns, and milder winters have all contributed to increased overwintering and earlier rust infections in the growing season [12]. Likewise, stripe rust populations have adapted to higher temperatures, opening the door for disease spread to warmer areas previously considered unsuitable [45]. These climate shifts complicate disease predictability, and the incidence of extreme events may rise.

#### 6.4.2. Alternate Hosts and Their Contribution to Disease Dynamics

**Figure 4:**
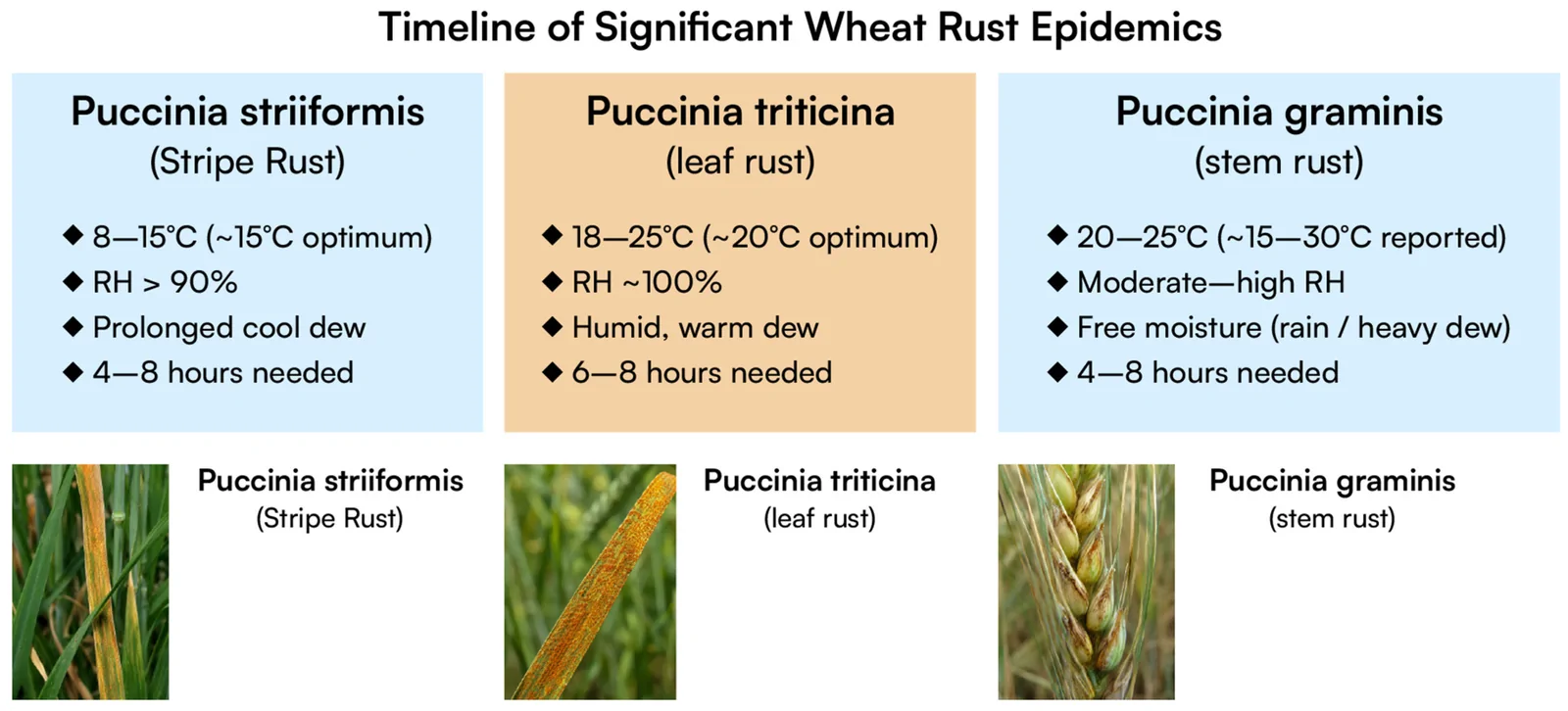
Environmental conditions conducive to wheat rust development.

Alternate hosts play an important role as rust pathogen reservoirs, allowing overwintering and genetic recombination (Figure 4). Among wheat rusts, stem rust relies most on alternate hosts. *Puccinia graminis* f. sp. *tritici* undergoes its sexual cycle on species of *Berberis*, with basidiospores infecting barberry leaves and producing pycniospores and aeciospores. Sexual recombination on barberry can produce new virulence combinations, greatly increasing the pathogen’s diversity and adaptive potential [40]. However, widespread eradication programs in North America and Europe have diminished the impact of this process by interrupting barberry’s sexual cycle and causing sharp declines in stem rust epidemics. In addition, the recovery of susceptible *Berberis* species in Europe, East Africa, and China has been associated with the reoccurrence of stem rust epidemics and elevated pathogen diversity, represented by the emergence of highly virulent races such as those within the Ug99 family [41]. The role of alternate hosts in leaf and stripe rust epidemiology is less well understood, but evidence is emerging. Experimental and field studies have detected traces of *P. triticina* on *Thalictrum* species as alternate hosts, but their involvement in epidemic development seems minimal in most areas [55,59]. So far, several *Berberis* and *Mahonia* species have been found to facilitate sexual recombination of *P. striiformis* f. sp. *tritici* in various regions of Asia, where alternate hosts can contribute to the development of new races under suitable ecological conditions [63]. In addition to actual alternate hosts, volunteer wheat plants and wild grasses serve as “green bridges” that facilitate rust pathogen survival between crop seasons. These reservoirs promote early-season infection and increase initial inoculum pressure, especially in areas with mild winters and continuous wheat cultivation [7]. In practical epidemiological terms, the two mechanisms contribute in distinct ways. Sexual recombination on alternate hosts is the principal source of new genetic diversity and novel virulence combinations, but its real-world impact is geographically restricted to regions where susceptible *Berberis* species coexist with wheat and complete the sexual cycle; it is also most significant for stem rust [40,41]. Asexual survival on green bridges, by contrast, rarely generates new races but is far more widespread and is the dominant driver of season-to-season carry-over and early epidemic onset for all three rusts, especially leaf and stripe rust in regions with mild winters and continuous wheat cultivation [7]. Thus, alternate hosts primarily shape the pathogen’s long-term evolutionary potential, whereas green bridges primarily govern the year-to-year inoculum supply that initiates epidemics. Stem rust, leaf rust, and stripe rust have pathogen-specific ecological preferences for temperature, moisture, and relative humidity. The color of urediniospores on wheat tissue indicates symptom expression and can visually distinguish epidemic intensities under optimal conditions [4,12,43].

### 6.5. Integrated Management Approaches for Wheat Rust Diseases

The important role of the environment and alternate hosts in wheat rust epidemiology emphasizes the need for integrated and pre-emptive management approaches. Monitoring weather, eliminating alternate hosts around wheat fields, controlling volunteer wheat, and adapting sowing dates can help minimize disease pressure. Integrating risk models based on climate and remote sensing with transboundary disease surveillance systems is increasingly improving our ability to predict potential threats early and respond accordingly. As climate change alters the suitability of environments for rust pathogens, studying and controlling these ecological drivers will be more critical to maintaining wheat production and global food security.

Integrated strategies, including conventional agronomics and modern biological, chemical, and digital techniques, are key to sustainable control of wheat rust diseases (Table 2). Economic crop losses occur because a single control method is continually overcome by the evolutionary capacity of Puccinia species and changing climate patterns. The ultimate goals of IDM are to reduce disease pressure, delay resistance breakdown, decrease environmental resource use, and secure sustainable production in the long term [13]. In this context, nanotechnology is emerging as an innovative component of integrated disease management, particularly through nano-enabled delivery systems and antifungal nanoparticles.

#### 6.5.1. Traditional Management Practices

Conventional rust control activities are key components of wheat disease management and have been implemented for generations under varying agro-ecological conditions. Cultural techniques such as rotation, sowing date, volunteer wheat management, and straw destruction reduce inoculum carry-over. Alternating wheat with non-host crops interrupts the rust cycle, whereas sowing early in some regions coincides with plant escape from periods of maximum infection [7,13,44]. Sanitation measures, such as removing alternate hosts (e.g., *Berberis* species), have played a successful role in managing stem rust epidemics in North America and Europe. Host resistance remains the most cost-effective and environmentally sound control method. Planting rust-resistant cultivars drastically mitigates disease severity at no extra operational expenses. However, resistance controlled by a single major gene is often not durable because the rust pathogen quickly evolves virulence to neutralize these genes [48]. Several popular cultivars (i.e., PBW 343 and Inqalab-91) exhibited high resistance at their initial stages but later proved to be susceptible as new rust races emerged. Although these methods are effective, the traditional approaches have relatively limited applicability. Monocropping, genotypic uniformity, and climatic variability have eroded the durability of resistance and increased the risk of epidemics, calling for modern strategies that complement current approaches.

#### 6.5.2. Novel Management Strategies

New methods for wheat rust control have recently been made possible by developments in plant pathology, microbiology, and precision agriculture.

##### Biological Control Approaches

Biological control relies on beneficial microorganisms to inhibit rust pathogens or induce host defense mechanisms. Microorganisms such as *Bacillus subtilis*, *Pseudomonas fluorescens*, and *Trichoderma harzianum* secrete antifungal metabolites, compete for resources, and trigger systemic resistance in wheat against plant pathogens [64]. Field studies in South Asia and Eastern Africa have demonstrated that seed treatment with beneficial microorganisms, microbial seed priming, and foliar applications might lower rust severity by 40–60% under moderate disease pressure [65].

Endophytic fungi and rhizobacteria have also been reported to increase wheat resistance to rust by stimulating defense-related responses and improving plant health [64,65]. Biological control, although environmentally friendly and suitable for organic agriculture, may have limited efficacy because of sensitivity to environmental factors or formulation challenges.

##### Chemical Control and Resistance Management

Chemical Control Fungicides: Fungicides remain an important tool for managing rust, especially when it is severe. Triazoles (e.g., tebuconazole, propiconazole), Strobilurins (e.g., azoxystrobin), and Succinate dehydrogenase inhibitors (SDHIs) are systemic fungicides used to protect wheat from rust infection [34,60,66]. Novel combination fungicides provide expanded spectrum control and enhanced efficacy against mixed rust infections. However, excessive, calendar-based fungicide applications have driven the development of fungicide-resistant rust populations and increased environmental and economic costs. To counter this, more resistance-management strategies, such as fungicide rotation, dose-rate optimization, and disease-forecasting-model-based variable dosing, are needed [13]. Recent advances also highlight the potential of nano-formulated fungicides, including nano-encapsulated and nano-emulsion systems that enable controlled and sustained release of active ingredients [67]. These formulations improve solubility, stability and targeted action of active ingredients, allowing lower application rates and reducing the likelihood of resistance development [19]. Moreover, certain nanoparticles have been reported to induce plant defense mechanisms, further contributing to disease suppression.

#### 6.5.3. Nano-Enabled Approaches for Wheat Rust Management

Nanotechnology is emerging as a promising tool for managing plant diseases, including wheat rusts. Nanoparticles (NPs), owing to their small size and high surface area, exhibit enhanced reactivity and improved interaction with microbial pathogens. Metal and metal oxide nanoparticles such as silver (AgNPs), copper (CuNPs), and zinc oxide (ZnO-NPs) have demonstrated strong antifungal properties through mechanisms including disruption of cell membranes [68,69], generation of reactive oxygen species, and inhibition of spore germination [70]. These multi-target actions reduce the likelihood of resistance development compared to conventional fungicides. Unlike conventional single-site fungicides, nanoparticles often act through multiple simultaneous mechanisms, thereby reducing selection pressure on pathogen populations and enhancing long-term disease control efficiency. In addition to their direct antimicrobial effects, nanoparticles can serve as carriers for controlled, targeted delivery of fungicides and biological agents. Nano-formulations improve the stability, solubility and bioavailability of active ingredients, enabling reduced application rates and minimizing environmental contamination [67]. This approach also supports environmental sustainability by reducing off-target effects, lowering chemical residues, and minimizing ecological toxicity [19].

Furthermore, certain nanoparticles have been reported to enhance plant defense responses by inducing systemic resistance mechanisms [71]. These interactions may involve modulating plant signaling pathways, including activating defense-related enzymes and enhancing oxidative stress tolerance, contributing to improved resistance against rust pathogens. These effects are largely governed by nano–bio interface interactions, where nanoparticles influence cellular and molecular processes within plant tissues and pathogen systems. Integrating nano-based formulations with precision agriculture tools may further improve application efficiency through site-specific delivery and optimized dosing strategies. Despite these advantages, challenges such as potential toxicity, regulatory concerns, and the cost of large-scale production remain. Therefore, further research is required to develop safe, cost-effective and field-applicable nano-based solutions for integration into wheat rust management systems. A promising future direction is combining nanotechnology with natural products such as plant extracts and essential oils, whose antifungal constituents can be nano-encapsulated to improve stability, solubility, and controlled release while reducing reliance on synthetic fungicides [33].

#### 6.5.4. Integration of Traditional and Novel Approaches

Combining traditional and modern methods offers the best option for durable control of wheat rusts. For example, resistant cultivars can be combined with threshold-based rather than calendar-based fungicide application guided by weather-driven forecasting; biocontrol seed treatments can be paired with adjusted sowing dates; and nano-formulated fungicides can be integrated with remote-sensing-based early detection to target only infected zones, reducing chemical inputs while maintaining control [13,26]. Any host plant resistance programs should consider biocontrol agents and judicious fungicide use, combined with cultural practice modification, to complement the resistant cultivars being developed. International programs, such as the Borlaug global rust initiative and national integrated pest management programs have shown that coordinated deployment of resistance genes, complemented by local monitoring and forecasting, has reduced rust impacts by 60–80% relative to conventional strategies [5]. Spore traps, weather-based risk models and satellite imaging are digital decision-support tools that improve precision and efficiency of integrated management systems [26]. Integrated disease management is beneficial not only because it increases the efficiency of disease control, but also because it lowers production costs, reduces environmental impact, and enhances resistance to a changing climate. Its success will largely depend on farmer awareness, access to resistant seed and advisory services, and support for research and extension systems.

A critical assessment shows that these approaches differ greatly in maturity and field readiness (Table 3). Host genetic resistance, cultural practices and fungicides remain the operational backbone of rust management, being mature and field-proven, although each is constrained by resistance breakdown, cost or partial efficacy. Marker-assisted and genomic selection have moved from experimental to established practice within well-resourced breeding programs, and remote sensing with AI-based forecasting is now operational within international surveillance networks. In contrast, the most novel biotechnologies—CRISPR-based genome editing, RNAi and host-induced gene silencing, and nano-enabled formulations—remain largely confined to experimental or early-stage settings; their limiting factor is generally not biological proof-of-concept but cost, regulation and delivery, particularly in the low- and middle-income countries that bear the greatest rust burden. This progression, spanning conventional breeding, wide hybridization from wild relatives, marker-assisted and genomic selection, and most recently genome editing, reflects a vast and still-expanding body of resistance-breeding research [48,72,73,74]. Recognizing where each approach currently stands is essential to prioritize realistic, deployable strategies rather than assuming emerging tools are ready to replace established ones.

### 6.6. Strategic Approaches to Enhancing Resistance Against Wheat Rust Diseases

Improving host resistance remains a key strategy for the sustainable management of wheat rusts. Given the dynamic nature of *Puccinia* spp. populations and frequent breakdowns in resistance, modern breeding strategies for rust resistance focus on durability, genetic diversity, and integrating conventional breeding with recent biotechnological tools. Durable resistance in the long term should bridge high disease resistance with good agronomic performance and environmental adaptation.

#### 6.6.1. Genetic Resistance to Wheat Rust

Genetic resistance to wheat rust is classified broadly as all-stage (seedling) resistance and adult plant resistance (APR). Most seedling resistance is conferred by single, race-specific genes that provide near-complete protection at young plant growth stages. Common examples include Lr15, Yr9, Sr17 and Yr27. Although it is powerful, only early generations of new virulent pathogen races can be resisted in the short term [5]. By contrast, adult plant resistance is typically polygenic (race-nonspecific) and tends to be expressed later in host development. APR is not to prevent infection, but rather to retard the progression of disease and limit spore production/epidemic development. Resistance genes such as Lr34/Yr18/Sr57, Lr46/Yr29, Sr2 and Lr67 have shown durable resistance under diverse environmental conditions to rust pathogens (Table 4) [51]. The Lr34 complex gene is also known to confer partial resistance to leaf rust, stripe rust, stem rusts, and powdery mildew by increasing basal defense responses. Recent genome-wide association studies and pangenome analyses have identified new resistance loci in landraces and wild relatives of wheat, emphasizing the importance of maintaining genetic diversity for resistance breeding [45,53,56].

#### 6.6.2. Breeding Strategies for Durable Resistance

Nowadays, plant breeders aim to pyramid genes, i.e., incorporate several resistance genes into a single cultivar to reduce the likelihood of resistance breakdown. By pyramiding seedling resistance genes with APR genes, breeders can provide both strong early protection and long-term durability [48]. Selected programs have accelerated from discovering a resistance QTL to commercial breeding for resistance using MAS and genome- or genotypic selection, which allow precise identification and tracking of resistance genes. Such strategies, which combine high-density SNP arrays and genomic prediction models, provide the opportunity to select superior genotypes before extensive field testing, thereby shortening breeding cycles and increasing selection intensity [48,73]. Nonetheless, resistance genes can pleiotropically affect important agricultural traits. Lr34, for instance, has been linked to leaf tip necrosis and impaired photosynthetic efficiency under some stress conditions. Hence, breeders should carefully balance disease resistance with yield potential, grain quality, and environmental adaptation interval [63]. Participatory breeding has also increased and is a key approach to involve farmers in selection so resistant varieties are better acclimatized to local conditions and more easily adopted by farming communities.

#### 6.6.3. Biotechnological Innovations in Wheat Rust Resistance

**Figure 5:**
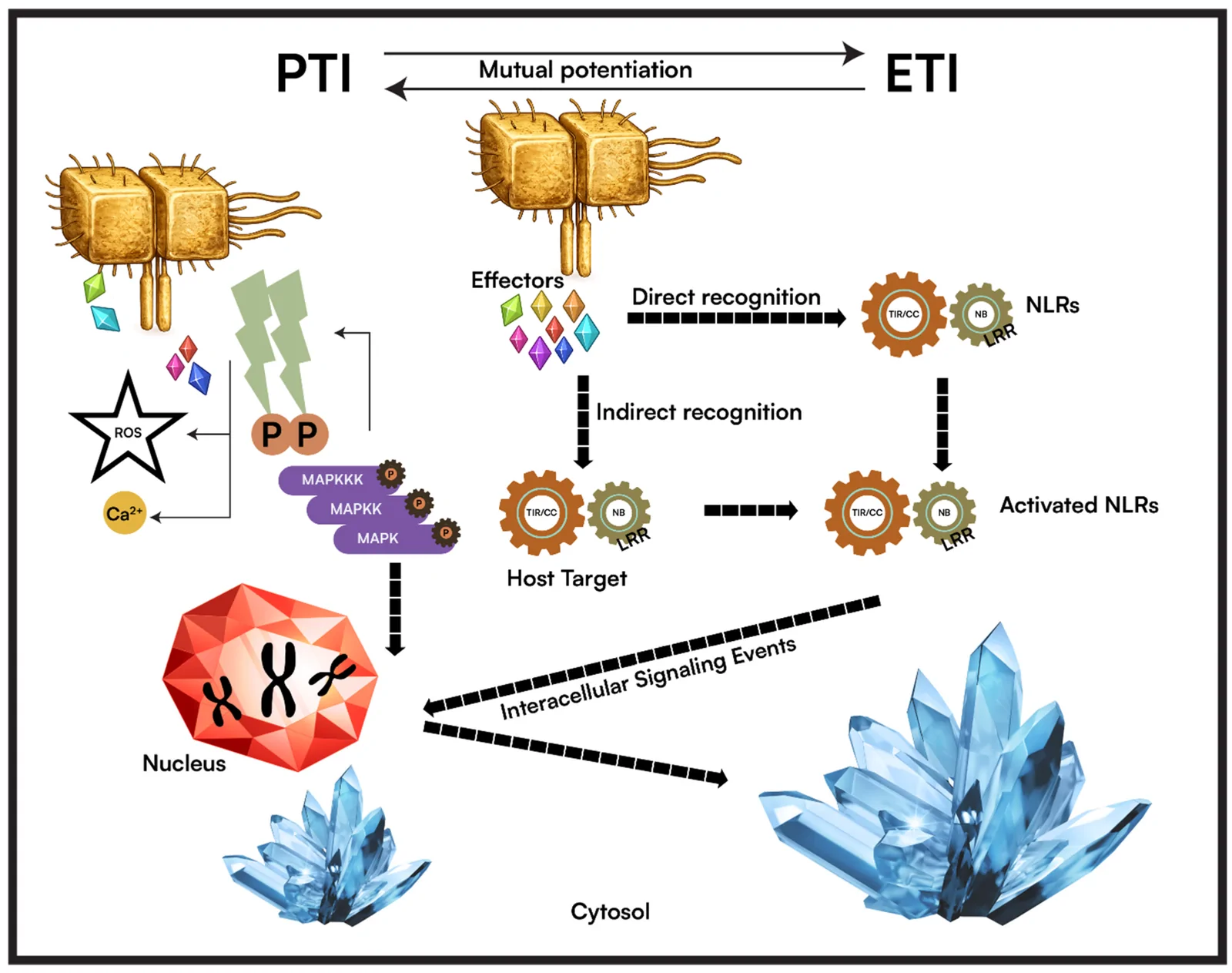
The genetic resistance mechanisms illustrate PTI and ETI layers of plant [innate] immunity.

Biotech has paved the way for improved resistance to wheat rusts by allowing specific modification of host and pathogen genes as needed. CRISPR/Cas9-mediated genome editing is a valuable strategy to increase resistance by knocking out susceptibility genes that promote pathogen infection. For example, modification of susceptibility genes has been shown to enhance resistance to certain fungal pathogens [74]. RNAi technology has also been applied to silence important fungal genes, including avirulence and effector genes such as AvrSr50 and AvrLr19, leading to rust suppression. Host-induced gene silencing (HIGS) is a potentially effective strategy for durable resistance, particularly against rapidly evolving rust populations [75]. Moreover, the resistance gene pool in wheat has been broadened by introgressing resistance genes from wild relatives, including *Aegilops tauschii* and *Thinopyrum elongatum*. These sources supply new alleles missing from current elite cultivars and are important for long-term resistance breeding [72]. Plant immunity against rust operates through two interconnected layers (Figure 5). In pattern-triggered immunity (PTI), cell-surface pattern-recognition receptors detect conserved pathogen-associated molecular patterns and activate basal defenses that restrict initial colonization. Successful pathogens secrete effector proteins that suppress PTI; in effector-triggered immunity (ETI), intracellular nucleotide-binding leucine-rich repeat (NLR) resistance proteins recognize these effectors, either directly or by guarding their host targets, and trigger a stronger, often hypersensitive response that halts pathogen spread. Most race-specific rust resistance genes encode NLR proteins acting through ETI, whereas several durable adult-plant resistance genes confer quantitative, broad-spectrum protection through distinct, non-NLR mechanisms [51,76] (Figure 5).

#### 6.6.4. Toward Durable and Sustainable Resistance

Sustained resistance will depend on an integrated approach to genetic diversity, regional deployment of resistance genes and ongoing pathogen vigil. When different cultivars with different resistance profiles are grown in space, selection pressure on pathogen populations becomes diverse [3,5]. Global diagnostic toolkits such as the Wheat Rust Toolbox and global monitoring systems provide the capacity to detect new phenotypes quickly and to guide breeding priority setting for surveillance. Together with modern agronomy, biotechnology, and climate-smart practices, these strategies provide a robust protocol for safeguarding wheat against future rust epidemics.

## 7. Future Prospects in Wheat Rust Management

**Figure 6:**
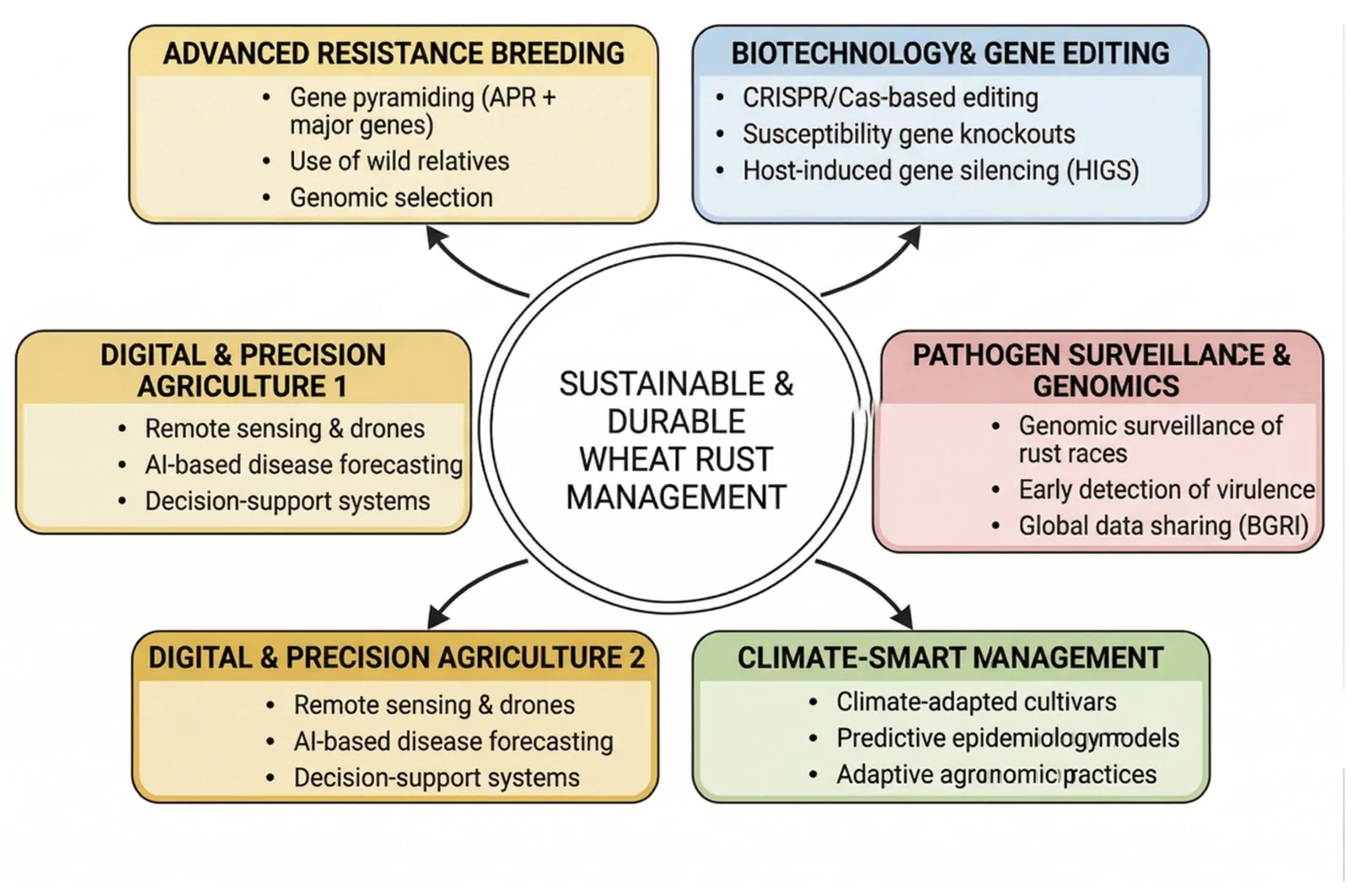
Future directions in research and management of wheat rust.

In our assessment, the future control of wheat rusts will depend on combining new technologies with strong partnerships across disciplines and continents. Because rust pathogens evolve rapidly in response to changing climatic and agronomic conditions, a proactive, predictive approach is essential to preserve global wheat productivity. Promising platforms for durable, sustainable control include resistance breeding for durable resistance, biotechnology and gene editing for broad-spectrum protection, pathogen-genomics-based surveillance, digital and precision-agriculture tools, and climate-smart management practices (Figure 6). This review highlights several tensions and gaps that we regard as priorities for the field. First, a persistent mismatch remains between the rapid pace of pathogen evolution and the slower cycle of resistance-gene discovery and deployment; addressing it will require pre-emptive gene stacking guided by real-time virulence surveillance rather than reactive, single-gene breeding. Second, the most novel technologies, genome editing, RNAi and host-induced gene silencing, and nano-based formulations, remain concentrated in experimental settings, and their translation is constrained less by biological proof-of-concept than by cost, regulatory uncertainty and delivery infrastructure, particularly in the low- and middle-income countries that bear the greatest burden. Third, surveillance capacity is unevenly distributed worldwide, leaving several major wheat-growing regions without the early-warning infrastructure on which all other interventions depend. Finally, the literature still contains unresolved questions—including the real-world epidemiological weight of sexual recombination on alternate hosts versus asexual carry-over on volunteer hosts, and the durability of newly characterized adult-plant resistance loci under diverse climates—that warrant coordinated, multi-region investigation. Progress will therefore depend not only on scientific advance but on aligning innovation with equitable delivery and sustained international data-sharing.

### 7.1. Emerging Technologies for Monitoring and Managing Wheat Rust

Technology developments, including digital surveillance and precision agriculture, are reshaping wheat rust monitoring. Remote sensing technologies, such as multispectral and hyperspectral satellite imagery, can enable detection before symptoms become visible on plants [52,54]. Vegetation indices (e.g., NDVI) and disease-specific spectral signatures are also used to detect infection hotspots and target interventions [15,54]. Machine learning and artificial intelligence (AI) models have recently made major strides in incorporating weather data, crop phenology, and historical disease reports to produce real-time risk alerts. Regional rust distributions can be tracked in near real time on platforms supported by international organizations, enabling early warning and rapid response [26]. Such tools reduce unnecessary fungicide applications, lowering production costs and environmental impact. From a molecular perspective, genome surveillance of rust populations is emerging as a key component of disease control strategies. High-throughput sequencing enables the rapid characterization of new virulence patterns and provides information for resistance deployment strategies. By linking pathogen genomics with host resistance response information, breeders and policymakers can predict, or at least anticipate, when resistance may breakdown before an epidemic occurs at a large scale [4]. Alongside digital and genomic tools, nanotechnology is expected to play a significant role in future crop protection systems. The development of smart nano-delivery systems, nano-sensors for early and real-time disease detection [19,77], and environmentally safe nano-fungicides offers promising avenues for precision and sustainable wheat rust management (Figure 6).

### 7.2. Climate Change Adaptation Strategies

Climate change is likely to exacerbate wheat rust threats through changes in temperature regimes, rainfall patterns and quantity, and rust pathogen overwintering survival (Table 5). Modeling suggests northward and uphill movement of stripe rust and leaf rust, as well as an elevated risk of stem rust in historically marginal areas [3,12]. Including climate projections in breeding programs can produce wheat varieties that are resistant to rusts and tolerant of heat and drought stress. Selection sensitivity is important for developing cultivars with multi-trait resilience and yield stability in susceptible areas [5]. Climate-smart disease management practices are imperative for mitigating these challenges. These include:

- Adjusting sowing dates: shifting planting windows so that the most susceptible crop stages avoid the periods of peak inoculum and favorable infection conditions is expected to reduce early epidemic establishment and lower the number of infection cycles per season.
- Deploying region-specific resistance gene combinations: matching the resistance genes in deployed cultivars to the virulence profile of the local pathogen population and rotating or combining them across regions is expected to slow resistance breakdown and extend the useful lifespan of resistance genes.
- Improving soil health, balanced nutrition, and diversity: maintaining optimal plant nutrition and using varietal mixtures and landscape-level genetic diversity to strengthen crop resilience and diversify selection pressure on the pathogen is expected to reduce epidemic severity and stabilize yields under variable climatic conditions.

**Table 5:** Climate change drivers influencing wheat rust epidemiology.

Climate Factor	Disease Impact
Warmer winters	Increased overwintering
Humidity shifts	Extended infection period
Rainfall variability	New outbreak zones
Temperature rise	Stripe rust expansion

### 7.3. Feasibility and Barriers to Adoption of Advanced Technologies in Low- and Middle-Income Countries

While genome editing, nano-enabled agrochemicals, and AI-driven surveillance offer considerable promise, translating them from research settings to field-level impact faces substantial economic, logistical, and regulatory barriers, particularly in low- and middle-income countries (LMICs) that bear the greatest rust burden. Economically, the high development and production costs of CRISPR-edited cultivars and engineered nanomaterials, combined with limited public breeding budgets and weak private-sector investment, restrict affordability for smallholder farmers [14]. Logistically, deploying nano-formulations and precision-agriculture platforms depends on input-supply networks, appropriate storage, mechanization, and digital infrastructure that are often lacking in rural wheat-growing regions. From a regulatory standpoint, many LMICs have not yet established clear biosafety frameworks for gene-edited crops or risk-assessment protocols for agricultural nanomaterials, creating uncertainty that delays approval and adoption. Fragmented extension services and low farmer awareness further constrain uptake even where technologies are available. Addressing these barriers will require targeted public investment, region-specific regulatory harmonization, capacity building, and public–private partnerships, so that advanced rust-management technologies do not remain confined to well-resourced systems but reach the farmers most exposed to epidemic risk.

### 7.4. International Collaboration and Policy Support

Given the transboundary nature of wheat rust diseases, international cooperation remains a key element in their management. Coordinated international efforts, including the Borlaug Global Rust Initiative and global cereal rust monitoring networks, enable germplasm exchange, standardized race phenotyping and joint surveillance across countries [5,27,32]. These efforts will ensure that new threats, such as novel Ug99 variants and aggressive stripe rust lineages, are detected quickly. Capacity building in developing countries is essential. Enhancing national surveillance systems, facilitating access to resistant seed, and expanding extension services are important measures to affect long-term reductions in vulnerability among smallholder farmers. Enabling policies that promote open data sharing, investment in public breeding programs, and sustainable use of inputs are necessary to sustain long-term successes. In our view, predictive genomics combined with AI-assisted scouting and participatory breeding constitute a way forward for developing resilient, low input wheat production systems under biological and climatic pressures.

## 8. Conclusions

Wheat rust diseases remain among the most destructive and constantly evolving crop diseases, threatening global food security because of their rapid evolution, high capacity for long-distance dispersal, and dependence on climatic conditions. Rust pathogens have persisted despite decades of improvements in breeding and disease control, evolving to defeat resistance genes and undermine conventional management tactics. This review makes clear that no single strategy is enough for sustainable control of wheat rusts. Established practices, such as crop rotation, sanitation, and resistant cultivars, provide a strong foundation but are increasingly threatened by resistance breakdown and climate variability. The way forward lies in integrated, systems-based management that combines conventional agronomy with modern biotechnology, digital monitoring, and climate-smart methods. Strategically using different resistance genes in combination, constant monitoring, and close international cooperation is key to slowing resistance erosion and avoiding massive epidemics. After all, adding rust disease protection to wheat producers’ arsenal of pest management tools is not just a technical challenge but also an economic and social/policy imperative. Critically, these scientific advances will translate into durable food security only if equitable delivery systems put resistant cultivars, affordable inputs, diagnostic tools, and advisory services within reach of resource-poor farmers, particularly smallholders in low- and middle-income countries where rust epidemics exact the heaviest toll. Without such delivery mechanisms, even the most sophisticated technologies risk widening, rather than closing, the gap between well-resourced and vulnerable wheat-producing regions. The need to invest in resilient and equitable wheat systems will therefore become increasingly important for securing stable yields, protecting farmer livelihoods, and sustaining global food and nutritional security as climate change accelerates and pathogens evolve.

## Figures and Tables

**Table 1:** Comparative biological and epidemiological features of major wheat rust diseases.

Feature	Leaf Rust	Stripe Rust	Stem Rust
Pathogen	*Puccinia triticina*	*P. striiformis* f. sp. *tritici*	*P. graminis* f. sp. *tritici*
Preferred Temperature	15–22 °C	9–15 °C	20–30 °C
Infection Site	Leaves	Leaves/spikes	Stems/spikes
Epidemic Nature	Chronic	Explosive	Catastrophic
Alternate Host	*Thalictrum* spp. (rare)	*Berberis*/*Mahonia* (regional)	*Berberis* spp.
Yield Loss Potential	10–30%	20–70%	up to 100%

Note: The preferred temperature ranges shown represent historically optimal conditions for each pathogen. These ranges are currently shifting under climate change; in particular, *Puccinia striiformis* f. sp. *tritici* now includes high-temperature adapted races that cause severe epidemics well above its traditional 9–15 °C range.

**Table 2:** Integrated management strategies for wheat rust diseases.

Approach	Method	Benefit
Cultural	Crop rotation	Reduce inoculum
Biological	PGPR *Trichoderma*	Induced resistance
Chemical	Triazoles	Immediate control
Genetic	Resistant cultivars	Sustainable
Digital	Remote sensing	Early detection

**Table 3:** Maturity and field-readiness of wheat rust management approaches.

Approach	Development Stage	Field-Proven?	Key Limitations	Key References
Resistant cultivars (major/seedling genes)	Mature	Yes, but often short-lived	Rapid resistance breakdown	[5,48]
Adult-plant/pyramided resistance	Mature	Yes	Longer breeding cycles; possible pleiotropic effects	[51,73]
Cultural practices (rotation, sowing date, sanitation)	Mature	Yes	Partial control; land and labour constraints	[7,13,44]
Chemical fungicides	Mature	Yes	Cost, resistance evolution, environmental impact	[13,34,66]
Marker-assisted/genomic selection	Established	Yes, in advanced programmes	Requires infrastructure and reference data	[48,73]
Biological control agents	Emerging	Partly; variable field efficacy	Environmental sensitivity, formulation	[64,65]
Remote sensing/AI-based forecasting	Emerging	Yes, within surveillance networks	Data gaps; limited access in LMICs	[26,54]
CRISPR/genome editing	Experimental	Not yet at field scale	Regulatory and delivery barriers	[74]
RNAi/host-induced gene silencing (HIGS)	Experimental	Proof-of-concept only	Stability, delivery, regulation	[75]
Nano-enabled formulations	Experimental–early	Limited field data	Toxicity, cost, scale-up, regulation	[19,67]

**Table 4:** Major types of genetic resistance against wheat rust diseases.

Resistance Type	Genetic Basis	Durability	Example Genes
Seedling Resistance	Major genes	Low	Yr9, Sr17
Adult Plant Resistance	Polygenic	High	Lr34, Sr2
Pyramided Resistance	Gene stacking	Very High	Yr + APR combos

## Data Availability

The raw data are available within the article.
